# Attenuation-based ultra-low-dose lung computed tomography at 0.1 mSv to 0.3 mSv effective dose in children

**DOI:** 10.1007/s00247-025-06503-z

**Published:** 2026-01-19

**Authors:** Max-Johann Sturm, Christian J. Kellenberger, Franco Rupcich, Sebastian Tschauner, Michael Zellner

**Affiliations:** 1 Department of Diagnostic Imaging, University Children’s Hospital Zurich, Lenggstrasse 30, Zurich, 8008 Switzerland; 2 Institute for Diagnostic and Interventional Radiology, University Hospital Würzburg, Würzburg, Germany; 3 Children’s Research Centre, University Children’s Hospital Zurich, Zurich, Switzerland; 4 GE Healthcare, Waukesha, USA; 5 Department of Radiology, Medical University of Graz, Graz, Austria

**Keywords:** Children, Multidetector computed tomography, Paediatric imaging, Paediatric lung, Radiation dose

## Abstract

**Background:**

Radiation dose reduction is essential in paediatric lung computed tomography (CT). Advances in energy-integrating detector CT and deep-learning reconstruction may enable ultra-low-dose imaging comparable to photon-counting CT.

**Objective:**

To evaluate the radiation dose and performance of an ultra-low-dose lung CT protocol using a wide-detector energy-integrating CT system in paediatric patients, focusing on effective radiation dose and diagnostic image quality.

**Materials and methods:**

A total of 277 low-dose lung CT scans from 106 paediatric patients (age range, 113 days to 17.85 years) were retrospectively analysed. All scans were acquired in axial mode using a 256-slice-multidetector CT scanner with deep learning image reconstruction and attenuation-based Auto Prescription. Radiation dose parameters, including volume CT dose index, dose-length product, size-specific dose estimate, and effective dose, were calculated. Signal-to-noise ratio and contrast-to-noise ratio were assessed in standardised anatomical regions. Patients were stratified by age, and statistical analysis was conducted to evaluate dose trends and image quality metrics.

**Results:**

There were significant differences between all age groups for all dose parameters (Kruskal–Wallis test, *P*<0.05). The median effective dose increased with age, ranging from 0.12 mSv (interquartile range (IQR) 0.09–0.14 mSv) in the 0–5-year group to 0.23 mSv (IQR 0.21–0.25 mSv) in adolescents aged 15 years to <18 years. Contrast-to-noise ratio and signal-to-noise ratio exhibited age-dependent variation with a small increase in older age groups. One-sided non-inferiority testing demonstrated that the signal-to-noise ratio and contrast-to-noise ratio in the youngest age group (0–5 years) were not significantly inferior to those in the ≥15-year group (*P*<0.05). All examinations were deemed diagnostically sufficient by board-certified paediatric radiologists. Non-disruptive artefacts such as cardiac motion and step artefacts occurred frequently but did not impair interpretation.

**Conclusions:**

Ultra-low-dose lung CT using wide-detector energy-integrating CT with deep-learning image reconstruction allows for routine diagnostic imaging in children at radiation doses ranging from 0.12 mSv to 0.23 mSv, comparable to those reported for newer photon-counting CT systems. This approach provides a robust, clinically viable strategy for minimizing radiation exposure while maintaining diagnostic image quality.

**Graphical abstract:**

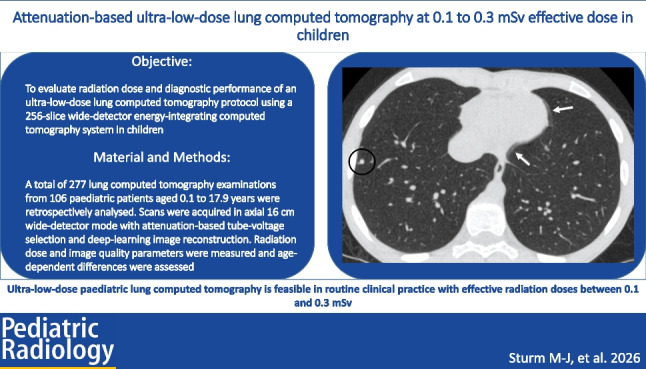

**Supplementary Information:**

The online version contains supplementary material available at 10.1007/s00247-025-06503-z.

## Introduction

Computed tomography (CT) is a widely used imaging modality that utilises ionizing radiation and is increasingly employed worldwide due to advantages such as broad availability and rapid acquisition times [1, 2]. However, CT scans account for the majority of medical radiation exposure globally, raising concerns about potential long-term risks [3].

In children, the benefits of contemporary multidetector CT — such as shorter scan times enabled by wider detector coverage, which also reduces the need for sedation [4] — must be carefully weighed against the potential risk of radiation-induced malignancies [5–9]. In accordance with the “as low as reasonably achievable” principle, radiation protection is a key priority in paediatric imaging [10]. Over the past years, initiatives such as “Image Gently” have enhanced awareness and promoted dose optimisation strategies in paediatric CT imaging [11, 12].

Recently, a remarkable reduction in radiation dose has been achieved using conventional energy-integrated detectors, especially for paediatric low-dose lung CT [4, 13–15]. Dose reduction in CT can be achieved through various approaches, such as x-ray beam hardening with e.g. tin filters [16], tube voltage modulation (e.g. attenuation-based) [17, 18], and advanced image reconstruction techniques such as iterative reconstruction and artificial intelligence-based approaches [17, 19–22]. Additionally, further dose reduction may be achievable with novel detector technologies, such as photon-counting CT [23–25]. Another strategy for dose optimisation is the use of axial scan mode with extended detector coverage in multidetector CT, allowing most of the paediatric chest to be captured in a single rotation. Compared with helical scanning, this reduces radiation dose and minimises table movement [26–28].

The objective of this study is to evaluate the effective radiation dose and experience of an ultra-low-dose CT protocol using attenuation-based tube voltage selection and axial scan mode on a 256-slice multidetector CT after three consecutive years of routine clinical use.

## Material and methods

### Patients

**Fig. 1:**
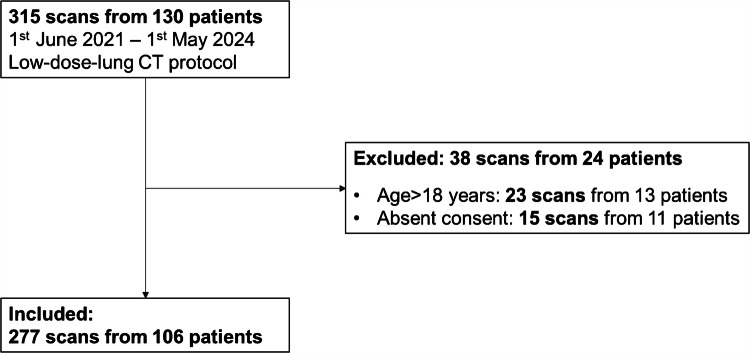
Flowchart illustrating the patient inclusion process. *CT* computed tomography

A retrospective analysis was conducted on 277 non-contrast-enhanced low-dose lung CT scans from 106 patients acquired between June 1, 2021, and May 1, 2024, using a 256-slice multidetector CT scanner (Revolution CT, GE Healthcare, Chicago, IL) at a tertiary care children’s hospital. All patients <18 years who underwent routine clinically indicated low-dose lung CT and who had provided written consent for evaluation of their medical data were included. Initially, 315 chest CT scans from 130 patients were considered for review. Of these, 38 scans from 24 patients were excluded. A flowchart of included and excluded cases is provided in Fig. 1. The following exclusion criteria were applied: patients older than 18 years at the time of examination (23 scans from 13 patients) and absence of consent (15 scans from 11 patients).

### CT acquisition and image reconstruction

All scans were acquired on a 256-slice multidetector CT using an attenuation-based Auto Prescription low-dose lung CT protocol in axial wide-detector mode, with deep learning image reconstruction (TrueFidelity DL, GE Healthcare) and the highest degree of noise reduction. The attenuation-based Auto Prescription approach selects the tube voltage according to attenuation information derived from the scout image, acquired with a fixed tube current of 10 mA and a fixed rotation time of 0.28 s. Patient attenuation was first measured by the scanner’s attenuation-based Auto Prescription algorithm and converted to anteroposterior and mediolateral dimensions. These dimensions were then converted to water-equivalent diameter using the conversion factors of Burton et al. [29]. Patients were subsequently automatically assigned to predefined tube-voltage intervals (80–140 kV) based on the water-equivalent diameter. Protocol details are summarised in Table 1.

**Table 1:** Technical and scan parameters of the low-dose lung computed tomography protocol with attenuation-based Auto Prescription used

Low-dose lung protocol	
**Technical parameters**	
CT scanner Detector type Detector rows Detector length Scan mode Slice collimation Tube voltage (range) Tube voltage (mean+SD) Tube current Rotation/exposure time Tube current - time Kernel Contrast agent Image reconstruction Pixel matrix Reconstructed slice thickness	GE® Revolution Energy-integrated-detector 256 16 cm Axial 0.625 mm 80–140 kV 116.8 kV (±16.1 kV) 10 mA 0.28 s 2.8 mAs Standard Non-contrast enhanced DLIR high 512×512 0.625 mm
**Auto Prescription derived diameter**	**Tube voltage**
<30 cm 30-<40 cm 40-<50 cm >50 cm	80 kV 100 kV 120 kV 140 kV

*CT* computed tomography, *DLIR* deep learning image reconstruction, *SD* standard deviation

### Signal-to-noise ratio and contrast-to-noise ratio

Signal-to-noise ratio and contrast-to-noise ratio as fundamental objective image quality parameters were measured by manually placed circular regions of interest in three different localisations: trachea and the liver. A minimum region of interest area of 5,000 pixels was used. Each measurement was performed three times on thin axial lung window slices (0.625 mm) to obtain the mean Hounsfield unit value and its standard deviation. Based on these parameters, signal-to-noise $\langle =SNR= \frac{\text{MEAN tissue}}{SD\text{ tissue}} ;\, SNR\text{ trachea}= \frac{\text{MEAN air}}{SD\text{ air}} \rangle$ and contrast-to-noise $\langle =CNR= \frac{\text{MEAN liver }-\text{MEAN air}}{SD\text{ air}}\rangle$ ratios were calculated with the standard deviation of air in the trachea representing image noise.

### Dose estimates

Computed tomography dose indices (=CTDIvol, calibrated using a 32-cm phantom, mGy) and the dose-length products $\langle =DLP= CTDIvol *\text{ scan length in cm }(\mathrm{mGy}*\mathrm{cm})\rangle$ were collected from the digital imaging and communications in medicine dose reports. In order to calculate the effective chest diameter, the anteroposterior diameter was measured manually in thin axial lung window slices (0.625 mm) at the level of the heart ventricles, as a straight line between the sternum and a spinal process of the thoracic spine. The mediolateral diameter was measured on the scout topograms as a horizontal line beneath the scapulae. Calculations of the size-specific dose estimates (mGy) and the effective doses (mSv) were based on the effective diameters in combination with the size-dependent conversion factors, published by the American Association of Physicists in Medicine [30] and according to Romanyukha et al. [31].

### Clinical history and indication

Information on clinical history and the indication of the scan were collected in the picture archiving and communication system (Sectra, Linköping, Sweden) and in the radiology information system (Cobra Software AG, Arlesheim, Switzerland).

### Statistical analysis

Collected data were analysed using SPSS Statistics, version 26 (IBM Corp., Armonk, NY). Normality of distribution was assessed using the Kolmogorov–Smirnov and Shapiro–Wilk tests. Gender differences were evaluated with the Mann–Whitney *U* test. Differences across age groups (0–5 years, 5–10 years, 10–15 years, and >15 years) were analysed using the Kruskal–Wallis and Mann-Whitney *U* tests. One-sided nonparametric non-inferiority tests were calculated using Statgraphics Centurion 19 (Statgraphics Technologies Inc., The Plains, VA) to assess our hypothesis that signal-to-noise and contrast-to-noise were not significant, and inferior in age group 0 to 5 compared to 15 plus years age groups. The upper equivalence ratio was set at 1.1, corresponding to a 10% non-inferiority margin relative to the older age group. Variables are reported as mean±standard deviation for normally distributed data, and as median with interquartile range (IQR) for non-normally distributed data.

## Results

### Patient characteristics and demographics

A total of 277 low-dose lung CT scans from 106 patients (mean age, 11.2±4.1 years) were included and analysed. Of these, 66 patients were male (63.3%) and 40 were female (37.7%). The comparison between male and female patients showed no statistically significant differences for any measured parameter (Mann–Whitney *U* test, all *P*>0.05). All patients underwent low-dose lung CT as part of routine clinical practice at a tertiary care university children’s hospital. All scans were diagnostically sufficient and interpreted by board-certified paediatric staff radiologists. The most common clinical indication was lung metastasis for tumour staging and follow-up. Demographic information and the respective indications for imaging are summarised in Table 2.

**Table 2:** Demographic characteristics, including computed tomography indications, and requirement for sedation or intubation

	Number (%)
**Sex** (***n***=106 patients)	
Female	40 (37.7%)
**Age at scan (years;** ***n*=277 scans)**	
0-<1 1-<5 5-<10 10-<15 15-<18	3 (1.1%) 15 (5.4%) 89 (32.1%) 115 (41.5%) 55 (19.9%)
**Indication**	
Lung metastasis Small airway disease Obstructive lung disease/asthma (Immunosuppression) infection (Recurrent) infection Restrictive lung disease Congenital/malformation Spontaneous pneumothorax Cystic fibrosis Other	207 (74.7%) 19 (6.9%) 11 (4.0%) 11 (4.0%) 8 (2.9%) 6 (2.2%) 3 (1.1%) 3 (1.1%) 2 (0.7%) 7 (2.5%)

For sex, individual patients were listed (*n*=106). For age, each computed tomography scan was considered separately, as patients underwent imaging at different ages (*n*=277)

The majority of scans (*n*=246) were acquired during inspiration, while 11 scans were performed during expiration. Sedation was required in 6 cases and intubation in 11, and in three examinations, patients were spontaneously breathing, particularly among younger age groups.

### Objective image quality and radiation doses

Contrast-to-noise ratio, signal-to-noise ratio, dose-length product, computed tomography dose index volume, size-specific dose estimate, and effective dose showed significant differences (*P*<0.05) across age groups, as determined by the Kruskal–Wallis test, with a consistent trend confirmed by the Jonckheere–Terpstra test. All mentioned parameters exhibited a consistent trend toward lower values in younger age groups (Table 3). Table 3 summarises the objective image-quality metrics and radiation dose parameters across the four age groups included in our study. The values shown reflect the results from this study population, while the lower lines of the table provide the relevant diagnostic reference levels [32].

**Table 3:** Objective qualitative parameters and radiation dose parameters divided by age groups, with dose reference levels shown in the lower rows

	Age group	CNR	SNR	DLP in mGy*cm	CTDIvol in mGy	SSDE in mGy	ED in mSv
**Current study**	0–5 years (*n*=18)	24.49 (20.78–29.39)	23.05 (19.48–27.72)	1.78 (1.43–2.51)	0.11 (0.11–0.14)	0.24 (0.22–0.28)	0.12 (0.09–0.14)
	5–10 years (*n*=89)	26.78 (24.35–30.19)	25.17 (22.78–31.13)	2.93 (2.71–4.40)	0.14 (0.14–0.21)	0.27 (0.25–0.34)	0.14 (0.13–0.17)
	10–15 years (*n*=115)	28.54 (25.85–32.18)	26.93 (24.36–30.47)	5.73 (5.03–6.85)	0.22 (0.21–0.22)	0.35 (0.33–0.38)	0.20 (0.17–0.23)
	>15 years (*n*=55)	30.69 (28.93–34.13)	28.92 (27.19–32.34)	6.94 (6.55–10.12)	0.22 (0.22–0.31)	0.36 (0.34–0.41)	0.23 (0.21–0.25)
**Dose reference level **[32]	0–1 years	NA	NA	76 (63–84)	3.6 (3.2–4.5)	4.8 (4.4–5.4)	NA
	1–5 years	NA	NA	70 (65–76)	2.5 (2.4–2.7)	4.1 (4.0–4.1)	NA
	5–10 years	NA	NA	93 (88–102)	2.9 (2.8–3.1)	3.9 (3.8–4.0)	NA
	10–15 years	NA	NA	210 (202–218)	5.8 (5.6–6.1)	5.9 (5.7–6.1)	NA
	>15 years	NA	NA	327 (313–339)	8.3 (8.1–8.6)	8.0 (7.8–8.2)	NA

Data is presented as the median with interquartile ranges

*CNR* contrast-to-noise ratio,* CT* computed tomography, *CTDI*_*vol*_ volume computed tomography dose index,* DLP* dose-length product, *ED* effective dose, *NA* not available,* SNR* signal to noise ratio,* SSDE* size-specific dose estimate

The contrast-to-noise ratio increased from a median of 24.49 (IQR, 20.78–29.39) in children aged 0–5 years to 26.78 (IQR, 24.35–30.19) in the 5–10 years group, 28.54 (IQR, 25.85–32.18) in the 10–15 years group, and 30.69 (IQR, 28.93–34.13) in those older than 15 years. The signal-to-noise ratio in the trachea showed a similar trend, rising from 23.05 (IQR, 19.48–27.72) in the 0–5 years group to 25.17 (IQR, 22.78–31.13), 26.93 (IQR, 24.36–30.47), and 28.92 (IQR, 27.19–32.34) with increasing age. Both the contrast-to-noise ratio and the signal-to-noise ratio in the 0-year to 5-year age group were demonstrated to be non-inferior to those older than 15 years, with one-sided nonparametric non-inferiority tests yielding a *P*-value=0.04 for the contrast-to-noise ratio and a *P*-value=0.04 for the signal-to-noise ratio.

**Fig. 2:**
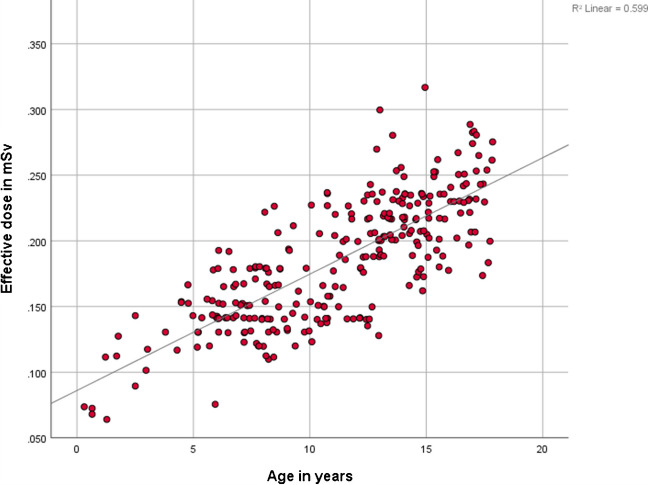
Age-dependent variation on effective dose of all lung computed tomography scans

The dose-length product increased steadily with age, from a median of 1.78 (IQR, 1.43–2.51) mGy·cm in the youngest group to 2.93 (IQR, 2.71–4.40) mGy·cm, 5.73 (IQR, 5.03–6.85) mGy·cm, and 6.94 (IQR, 6.55–10.12) mGy·cm in the older age groups, reflecting the larger scan coverage required in older children. The volume computed tomography dose index rose from a median 0.11 (IQR, 0.11–0.14) mGy in the 0–5 years group to 0.14 (IQR, 0.14–0.21) mGy, 0.22 (IQR, 0.21–0.22) mGy, and 0.22 (IQR, 0.22–0.31) mGy in the successive age groups, consistent with the need for higher tube output to penetrate larger body sizes. The size-specific dose estimate increased from a median 0.24 (IQR, 0.22–0.28) mGy in the youngest patients to 0.27 (IQR, 0.25–0.34) mGy, 0.35 (IQR, 0.33–0.38) mGy, and 0.36 (IQR, 0.34–0.41) mGy in older children and adolescents. The effective dose showed a gradual rise from a median of 0.12 (IQR, 0.09–0.14) mSv in children aged 0–5 years to 0.14 (IQR, 0.13–0.17) mSv, 0.20 (IQR, 0.17–0.23) mSv, and 0.23 (IQR, 0.21–0.25) mSv in the successive age groups (Fig. 2).

### Artefacts

**Fig. 3:**
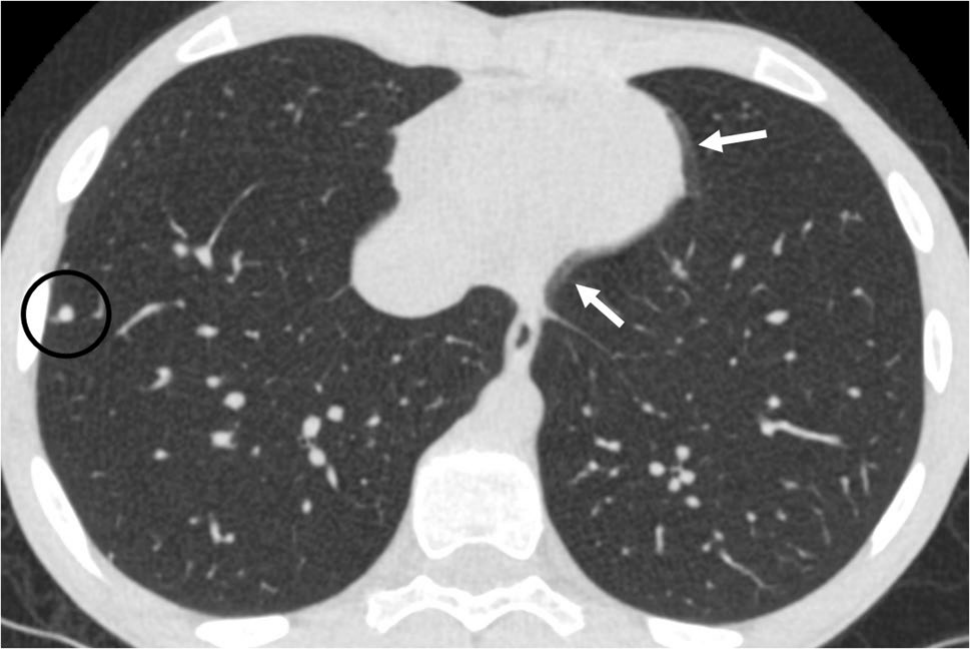
Representative axial lung computed tomography image of a 13-year-old boy, demonstrating cardiac pulsation artefacts (*white arrows*) and a lung metastasis (*black circle*). The examination was performed with a dose-length product of 5.73 mGy·cm, a volume computed tomography dose index of 0.22 mGy, a size-specific dose estimate of 0.36 mGy, and an effective dose of 0.22 mSv

All included scans met diagnostic quality standards and were reviewed by a radiologist. However, non-disruptive artefacts were frequently observed, without compromising diagnostic accuracy. Cardiac pulsation artefacts were consistently present across scans, as a result of the heart’s inherent rhythmic motion (Fig. 3). Due to the acquisition mode of axial scans utilizing a 16-cm detector length, step artefacts occurred when the thoracic length exceeded this range. On average, 1.26±0.56 step artefacts were required to cover the entire thoracic region in the included patients.

## Discussion

Radiation protection is crucial in paediatric imaging, particularly in paediatric CT. Therefore, we aimed to analyse the radiation doses achieved by the low-dose lung CT protocol established in 2021 over three consecutive years of routine clinical use. We demonstrated that an effective dose consistently below 0.3 mSv was maintained in routine clinical imaging in paediatric patients using a 256-row multidetector CT system with energy-integrating detector technology, reaching values as low as 0.09 mSv in the youngest patients.

These findings are in line with previous studies on low-dose lung CT protocols, which reported effective doses of approximately 0.2 mSv [26]. This study represents a large paediatric cohort on effective dose with energy-integrated detector CT, comprising 277 scans from 106 patients, following prior reports by Esser et al. (2007–2014; 1965 scans, 768 patients) [13] and Singh et al. (2007–2009; 328 scans) [33]. Our data confirm that the downward trend in effective dose for paediatric lung CT reported by Esser et al. has continued, reaching levels well below the 0.7–2.0 mSv achieved by 2014 [13]. Our low-dose lung CT protocol is based on a combination of a 16-cm coverage using an energy-integrated detector CT 256-row multidetector in axial acquisition mode, DLIR-techniques, and attenuation-based selection of tube current. The rationale for using axial scanning mode is to minimise radiation exposure time and table movement, as the majority or even the entirety of a paediatric thorax can be covered within a single gantry rotation, completed in 0.28 s. The benefits of reduced exposure time and radiation dose associated with this approach have been demonstrated in a randomised controlled trial conducted in an adult population [28]. Notably, alternative dose reduction strategies using helical acquisition mode with increased pitch have also proven to be feasible and effective. Comparable low effective doses of approximately 0.2 mSv have also been achieved using helical chest CT acquisitions with energy-integrated detector CT [14, 34] as well as with the novel photon-counting detector technology [23]. In the case of photon-counting detector CT, dose reduction is facilitated by optimised detector efficiency in combination with high-pitch acquisition. However, photon-counting detector technology remains relatively recent and limited in clinical availability, while energy-integrating detector CT continues to be the predominant modality in clinical practice worldwide. Nevertheless, given the novelty of photon-counting technology, combined with promising results from initial phantom studies [27] and ongoing advances in reconstruction techniques [35, 36], it is likely that even lower radiation doses will be achievable with photon-counting CT in the near future. Nevertheless, we would like to emphasise that in our study using energy-integrated detector CT, we observed doses comparable to those reported by Tsiflikas et al. using photon-counting detector CT, with a median size-specific dose estimate across all age groups of 0.34 mGy compared to 0.45 mGy reported by Tsiflikas et al. [23]. Furthermore, our CTDIvol values were substantially lower than those reported by Siegel et al. for both photon- and energy-integrated detector CT systems. In our cohort, CTDIvol medians ranged from 0.11 mGy to 0.22 mGy (IQR, 0.11–0.31), compared to a median CTDIvol of 0.41 mGy (IQR, 0.30–0.39) for photon-counting detector CT and 0.71 mGy (IQR, 0.68–0.79) for energy-integrated detector CT, as described by Siegel et al. [25]. Furthermore, our data fall within or below the median CTDIvol of 0.27 mGy reported for photon-counting detector CT by Krueger et al., and remain substantially below the energy-integrated detector CT group, which had a median of 0.39 mGy [37]. However, Zellner et al. demonstrated in a phantom study that low-dose chest CT with a CTDIvol of 0.07 mGy may be feasible utilizing a photon-counting detector CT [27]. Notably, the difference between the CTDIvol and size-specific dose estimate values in our study and the recommended dose reference levels (Table 3) is even more substantial. The size-specific dose estimate values in this study are approximately 20-fold lower than the corresponding dose reference levels [32], underscoring the magnitude of dose reduction achievable with this protocol.

The CNR ranged from 24.49 to 30.69 (IQR, 20.78–34.13), exceeding the initial values of 23.2±3.5 observed at a similar protocol implementation [26]. These findings highlight the robust diagnostic image quality achieved in our study.

Although this trend toward lower radiation doses in younger children has been observed in previous studies [13], Zellner et al. recently demonstrated that an attenuation-based Auto Prescription approach for cranial CT achieved a more balanced dose compared to an age-based protocol alone [38]. The dose reduction achieved with attenuation-based Auto Prescription in body imaging, including the chest, is supported by phantom studies [39, 40].

In this study, nearly 75% of scans were performed for oncologic indications, primarily for staging and follow-up of pulmonary metastases. Similarly, Esser et al. reported that most referrals originated from paediatric haematology and oncology, although pneumonia was the predominant indication in their cohort [41]. In contrast, our data show a clear predominance of oncologic over inflammatory indications, reflecting the growing use of lung magnetic resonance imaging for conditions such as pneumonia and cystic fibrosis [42–44]. This is underscored by the fact that only two patients with cystic fibrosis underwent chest CT during the 3-year study period (Table 2). However, CT remains the gold standard for imaging the lung parenchyma, especially for tumour assessment, and continues to play an essential role in paediatric oncologic imaging [45–47]. Especially in view of repeated imaging, optimising low-dose protocols to minimise cumulative radiation exposure is of great importance [48]. Over recent decades, efforts to reduce sedation and intubation in paediatric imaging have intensified [49]. A key strategy is minimising scan time through wide-detector CT systems capable of covering up to 16 cm of the thorax in a single 0.28-s rotation. The low sedation rate of <10% (26/277) in our cohort supports the feasibility of this approach in clinical routine, consistent with previous reports [4, 50]. Imaging and artefacts are inherently interconnected [51]. Two types of artefacts were commonly observed in our cohort of low-dose lung CT scans. First, cardiac pulsation artefacts were almost universally present due to inherent cardiac motion, which is a well-documented limitation across various imaging modalities, including low-dose lung CT [52]. Second, wide-detector axial acquisition can lead to step or stitch artefacts when the thoracic length exceeds the 16-cm detector coverage, requiring multiple acquisitions to be stitched together. These artefacts arise from variations in inspiratory depth between acquisitions and depend on patient compliance, ranging from minimal to pronounced.

A limitation of this study is its single-centre, retrospective design, which relied on routine clinical CT scans and associated radiation doses. As such, we could not control for variables like patient motion or minor technologist-dependent differences. The study was performed on a single scanner brand using one vendor’s deep learning reconstruction algorithm, which may limit generalisability to other platforms. Subjective image quality assessment was not performed, as it was deemed unnecessary: all scans were acquired in clinical routine, no repeat scans were required, and each examination was interpreted as diagnostically sufficient by a board-certified paediatric radiologist at the time of reporting. Furthermore, the adequacy of the protocol for producing diagnostic-quality images was previously validated in a phantom study.

## Conclusion

This study demonstrates that paediatric low-dose lung CT using energy-integrated detector systems is feasible in clinical routine, achieving effective doses of 0.1 mSv to 0.3 mSv. These values are comparable to those reported by photon-counting detector CT, which is not yet widely adopted in clinical practice. This highlights the substantial potential for dose reduction with current energy-integrated detector CT technology.

## Supplementary Information

Below is the link to the electronic supplementary material.

ESM 1 (59.8 KB SAV)

## Data Availability

Anonymized data supporting the findings of this study are available from the corresponding author upon reasonable request. In addition, Database 1 (SPSS file) has been uploaded as supplementary material and is available to reviewers during the peer review process.
